# CanMEDS Competencies in Family Medicine Residents: Can Criterion-Based Assessment Improve the Quality of Teacher Feedback?

**DOI:** 10.15694/mep.2021.000016.1

**Published:** 2021-01-19

**Authors:** Caroline Simard, Luc Côté, Laurie de Bruyn, Miriam Lacasse

**Affiliations:** 1 Université Laval

**Keywords:** Computed-based feedback, feedback, criterion-based assessment, family medicine residency.

## Abstract

This article was migrated. The article was marked as recommended.

**Background:** The Université Laval family medicine program has developed an innovative computerized tool called the criterion-based Competency Assessment Tool (CAT), currently undergoing validity assessment.

**Methods:** This study followed a qualitative design assessing written comments collected in the assessment reports from the cohorts before and after the implementation of the CAT (n _pre_ = 200, n _post_ = 200) in order to ascertain the tool’s consequence validity. A deductive thematic content analysis was performed and pre- and post-implementation cohorts were compared.

**Findings:** Overall feedback quality does not appear to have changed between cohorts. When analyzing CanMEDS roles separately, each is covered more often, but related comments appear to be less specific. The new report also seems to enable the teacher to tell more with the same number of words.

**Conclusions:** Perhaps since the items are complete, exhaustive, and detailed enough to be self-explanatory, the tool helps the teacher to cover a wider area of competencies without the need to add many details with narrative comments. Consequence validity does not seem to have been substantially affected by changes in the family medicine resident’s competency assessment, but the results do not support the contention that comment quality has improved either.

## Introduction

Feedback is the cornerstone of medical education or at the very least, it is central to improving a resident’s performance. When feedback is delivered in a ‘state of the art’ manner, its virtues can be very beneficial indeed. It especially helps the recipient regulate his or her learning through improved reflection ( Ericsson *et al*., 2006; van den Boom *et al*., 2004) and greater engagement ( Burgess and Mellis, 2015; Price, Handley and Millar, 2011). By reducing the “gap between what is understood and what is aimed to be understood” ( Hattie and Timperley, 2007, p. 82), feedback also helps the resident to detail his or her own strengths and limits, as well as what to learn and how it can be achieved ( Hattie *et al*., 2007; Norcini and Burch, 2007). Contemporary educational models place emphasis on the interactive aspect of the relationship between the feedback provider and its recipient and on the collaborative construction of knowledge ( Ossenberg, Henderson and Mitchell, 2019). Telio, Ajjawi and Regehr (2015) proposed the learner-centered approach of *educational alliance*, which corresponds to the learner’s perception of his or her trainee’s authenticity and commitment to a learning process, involving “seeking shared understanding of performance and standards, negotiating agreement on action plans, working together toward reaching the goals, and co-creating opportunities to use feedback in practice” (p. 612). Bing-You *et al*. (2018) used the metaphor of the “tango dance” to illustrate the complexity of the partnership between the clinical teacher and the student. In return, this educational dialogue encourages attitudes of feedback recognition, usage, and seeking in learners ( Bowen, Marshall and Murdoch-Eaton, 2017). In this context, while quality feedback contributes to educational alliance, the latter help foster adherence to what has been conveyed.

However, feedback quality is far from being uniformly satisfying ( Bing-You *et al*., 2018). When feedback fails to be constructive, and especially if clinical teachers’ comments are non-specific and non-descriptive, it can hinder learning or cause negative outcomes, such as uncertainty, questioning teachers’ credibility, a reduction of receptiveness or adherence, and mostly a decision to avoid seeking feedback ( Archer, 2010; Baron, 1988; Telio, Ajjawi and Regehr, 2015; van de Ridder *et al*., 2015).

The consensus is well established in medical education concerning what constitutes effective feedback, leading to several recommendations ( Archer, 2010; Boehler *et al*., 2006; Brehaut *et al*., 2016; Brinko, 1993; Canavan *et al*., 2010; Chur-Hansen and McLean, 2006; Ende, 1983; Ghazal, Gul and Khowaja, 2015; Jongekrijg and Russell, 1999; Lefroy *et al*., 2015; Molloy *et al*., 2018; Ossenberg, Henderson and Mitchell, 2019; Parkes, Abercrombie and McCarty, 2013; Saraf *et al*., 2014; Watling and Ginsburg, 2019).

• Feedback should first be relevant to the recipient. It should be criteria-based, linked to personal goals meaningful to the learner, related to well-defined goals and performance indicators, and explicit regarding whether the learner met the standards. In addition, the information should be factual and based on remediable behaviours. Advice should be future-oriented instead of dwelling on the past.
• Secondly, constructive feedback should be specific. Specific feedback is focused on tasks, concrete information, and behaviors. It should not be based on inferences, generalizations, judgements, or on the learner’s character.
• Thirdly, feedback should include pathways for improvement. Providing suggestions, limits, and omissions offers guidance and facilitates one’s performance review. Balancing both positive and negative comments is beneficial to feedback constructiveness, though the strict usage of the popular “feedback sandwich” technique (positive-negative-positive feedback; LeBaron and Jernick, 2000) does not appear always to be successful in increasing performance. Molloy *et al*. (2018) suggest instead to prioritize *feedback literacy* and help the learner develop his or her capacity to “anticipate emotion and manage emotions in relational activities, particularly when there are disparate perspectives on performance between the learner and the “other”, whether that is the teacher, peer, or patient” (p. 2). While attention should be focused on preserving self-esteem, feedback providers should also identify strengths and reinforce achievements, without, however, offering praise. Awareness should be raised concerning the tone of feedback, depending on whether it is presented in terms of reflection, praise, criticism, or suggestion. In the end, instead of focusing on a one-way “sandwiched” feedback or on the feedback delivery, the clinical teacher should focus on the interactive process and orient a dialogue toward both strengths and pathways for improvement in order to stimulate reflection and self-assessment.

However, the mere “mechanical” application of the aforementioned recommendations is also detrimental to educational alliance and adherence to feedback pathways. They should be conceived in the broader context of a dialogue and interactive process. In light of these recommendations, previous works investigated whether feedback was effective in improving physicians’ performance. Veloski *et al*. (2006) conducted a systematic review of 41 studies measuring physicians’ performance before and after they received feedback. Only 74% of studies ended with positive results in terms of performance. In a following meta-analysis, Mansouri and Lockyer (2007) retrieved 31 studies concerning interventions in continuing education aimed at improving physicians’ performance. Only one article included a written feedback intervention and the effect on knowledge and performance was not significant. Bing-You *et al*. (2017) reviewed articles about feedback for learners in medical education and only a few articles compared performance before and after feedback. Moreover, studies on feedback applied to medical education are even rarer, and most rely on less rigorous methodologies ( Bing-You *et al*., 2017). In the context of these partial or mitigated results, ongoing efforts must be made to better understand the virtues of feedback and especially the characteristics of constructive feedback, as well as how it can be influenced by assessment tools.

### The current study

Université Laval is one of three francophone faculties of medicine in the province of Quebec, Canada. Its family medicine residency program developed an innovative electronic tool, the criterion-based Competency Assessment Tool (CAT; Lacasse *et al*., 2017), in response to the new physician competency framework established by the Royal College of Physicians and Surgeons of Canada and the College of Family Physicians of Canada, namely the CanMEDS ( Frank, Snell and Sherbino, 2015).

This tool is based on rubrics that represent program expectations with regard to achievement of various CanMEDS-MF competencies in the course of residency and specifies the time intervals for each supervision level (or independence level). The assessed competencies and expected performance for each of these differ depending on the rotation and the training period. The CAT can suggest educational diagnoses and prescriptions based on the assessment result so as to support resident feedback and guide clinical faculties in their decisions on the rotation outcome. The CAT is further detailed in the Measures section.

The written feedback it comprises aims at helping students glean an accurate portrait of their progress throughout the program. In this context, it is essential to verify the quality of the feedback given and whether it has improved with the implementation of the new assessment tool.

## Methods

This cohort study compares the written comments on the assessment reports concerning residents’ competencies before and after implementation of the CAT. We used a mixed-methods convergent study design ( Sunderji and Waddell, 2018) to ascertain whether the CAT is associated with a change in the quality of written comments included in the resident assessment reports. The research was approved by the Université Laval ethics committee and the faculty direction.

### Data collection

Assessment reports were provided by the Université Laval family medicine residency program for the 2013-2015 pre-implementation cohort (n =1037) and the 2016-2018 post-implementation cohort (N = 1380). For comparison purposes, 200 reports per cohort were selected among those that included comments (N _pre_ = 1025, 98.84%; N _post_ = 1115, 80.80%). All reports identifying residents as “in difficulty” in the capstone were included (n _pre_ = 8, N _post_ = 25), and then the rest were chosen randomly, respecting a quota of 100 reports for residents completing the first year of residency (R1) and 100 reports for R2 (second year of residency) that is, for each cohort.

### Measures

The CAT is an online assessment tool comprising 34 competencies (indicators) grouped in seven roles: medical expert, communicator, collaborator, leader, health advocate, scholar, and professional. This new criterion-based competency assessment tool (CAT) takes into account both the program expectations in terms of competency development across time and the resident’s progress achievement. This summative instrument is generally used monthly or bimonthly by clinical teachers. As the CAT is an electronic-formatted tool, it helps clinical teachers in their assessment by adding examples of behaviours describing observable manifestations of the CanMEDS associated with each competency in the form of a notice that appears during a mouse hover. These examples aim to help clinical teachers in assessing the resident more objectively. The CAT also makes it possible to write comments about each CanMEDS role. Finally, it guides clinical teachers in determining the issue of the rotation, namely whether the resident passes, fails, is considered in difficulty, or is put on probation.

### Qualitative coding

Clinical teachers’ feedback in each assessment report was qualitatively and deductively coded independently by researchers (2013-2015 cohort: CS and LDB; 2016-2018 cohort: CS, LC, and LDB) based on the following three criteria described in the aforementioned literature:

***Relevance.*** Firstly, constructive feedback must be relevant, suggesting that the evaluated (and commented) elements are part of the assessment report or are directly related to it. In the current study, relevance was operationalized as follows: *The comment refers explicitly to the definition elements of the CanMEDS roles and to the assessment report’s content.*

***Specificity.*** Secondly, constructive feedback must be specific. It must also be based on facts or actions and anchored in descriptive data, instead of interpretations and presumed intentions. In this study, specificity was operationalized as follows: *The comment refers to specific facts or behaviors related to the resident’s performance aligned with CanMEDS roles rather than general and interpretative aspects.*

***Presence of pathways for improvement.*** Thirdly, feedback is constructive when areas of improvement are provided. To maximize feedback efficacy, the proposed pathways for improvement should ideally be accompanied by suggestions about how to achieve the desired improvement. In this study, the presence of pathways for improvement was operationalized as follows: *The report comments include at least one area of improvement, which can be mentioned as a specific educational prescription or as a limitation to be corrected.*

Relevance (abridged as “R”) and Specificity (abridged as “S”) were coded for each comment, while presence of Pathways for improvement (abridged as “PI”) was coded throughout each report. The PI score was generalized in this sense because pathways for improvement can be suggested intrinsically without necessarily being present in every comment and without undermining the quality of feedback received through the assessment report. We transformed qualitative data into quantitative data by attributing a score of “1” when the criterion was present in a given comment, and a score of “0” in its absence, resulting in a dichotomous variable.

As there are discrepancies between the number of competencies (and comments) as regards pre- and post-implementation reports, the two principal researchers (CS and ML) linked the pre-implementation comments for each CanMEDS role they referenced (discrepancies revised by ML). Afterwards, three coders (CS, LC, and LDB) analyzed the presence/absence of R, S, and PI in the 30 first reports before meeting to discuss ambiguities and potential adjustments to coding. The process was repeated with 30 other reports. After the two calibration meetings, they independently revised the first 60 reports, and then analyzed the other 140. Subsequently, the 200 pre-implementation reports were independently analyzed by CS and LDB. For further analysis, Valence was also coded (i.e. if the comment is 0 = negative; 0.5 = neutral; or 1 = positive).

### Score computation

The initial analysis led to a Relevance and Specificity score for each CanMEDS role and each rater. Divergences between coders were identified. In the pre-implementation cohort, the first author revised both raters’ scores to determine the final score when a consensus was not reached. In the post-implementation cohort, because there were three raters, the final score was determined based on the majority of responses. In other words, the option (presence or absence) was established according to the highest number of votes (i.e. two versus one). To get only one Relevance and one Specificity score per report, average values were computed (the average of each role score). Afterwards, a feedback quality score (FQS) was computed through averaging the mean R, mean S, and PI. Hence, scores varied from 0 to 1 and were reported in percentages, thus facilitating interpretation.

### Quantitative data analysis

Inter-rater reliability was verified with intra-class correlation (two-way mixed, absolute agreement). Internal consistency was verified in order to obtain an interpretable feedback quality score derived from R, S, and PI. Chi-squared testing was performed to see whether there were differences in CanMEDS coverage between cohorts. Analysis of variance (ANOVA) was performed regarding pre-FQS (2013-2015) and post-FQS (2016-2018) to ascertain whether differences in feedback quality have emerged since implementation of the CAT, this being the main purpose of the study. To document the context of feedback quality, the three feedback quality criteria were correlated with comments’ valence and residents’ performance. Performance is first represented by CAS ( *competency acquisition score*, which is in simple terms a percentage of mastery in all the CanMEDS competencies), but this score is only available for the 2016-2018 cohort. The CAS has the advantage of making results uniform across stage types and of weighting competencies according to their relative importance for each stage. However, as this score is based on a very strict specific competency set for each stage, it does not consider partial success and it treats missing data as a failure. Its use being contextually appropriate, the CAS might engender bias in the current study. In this regard, we also correlated feedback quality criteria with percentage of success in competencies (PSC score), which could also be available for the 2013-2015 cohort. Both CAS and PSC vary from 0 to 1 and are based on the final competencies score; hence they should be somehow comparable. However, the PSC excludes missing data and average available data. Because scores vary from 1 to 3 in the 2013-2015 cohort and from 1 to 4 in the 2016-2018 cohort, they were recoded between 0 and 1 before being averaged. As concerns verifying whether feedback quality was different if the students experienced isolated competency achievement delays (if concerns were raised about their progress), or if they were classified as being in difficulty or as failures, inferential statistics could not be computed due to small sample sizes. Still, descriptive statistics were computed. Finally, as residents’ programs were grouped into six categories, we compared the three quality criteria among programs through ANOVA. When the equal variance assumption was not met, Welsh’s adjusted *F* was computed ( Liu, 2015; Tomarken and Serlin, 1986). Post-hoc analyses were performed using the Bonferroni correction, or in the case of unequal variance, using the Games-Howell test.

## Results/Analysis

Among the 400 reports analyzed, 131 involved male students’ (32.8%), and 263 involved female students, with 6 being gender unspecified (65.8%; with 26 reports missing data concerning a pair of residents in the post-implementation cohort = 1.5%). A total of 88 residents were represented in the 2013-2015 cohort reports (of 101 residents in the cohort), and 95 residents were represented in the 2016-2018 cohort reports (of 106 residents in the cohort).

In order to illustrate the criteria and their application, we provide in Table 1 a few examples from the CanMEDS role expressed as “medical expert” (freely translated from French to English by the authors), where some criteria are present or absent.

**Table 1.** Comments extraction to illustrate feedback quality criteria

Comments (with original French version)	R	S	PI
**Professional role**			
“Is constantly ready to learn. Good attitude, favourable to exchanges.” *“Se met en position d’apprentissage. Belle attitude favorable aux échanges.”*	1	1	1
“Adequate knowledge for the training level.” *“Connaissances adéquates pour le niveau.*	0	0	0
Knows her limits, but difficulties are too important for this rotation level.” *“Connait ses limites, mais limites trop importantes pour niveau de stage.”*	1	0	0
“Wants to learn, professional approach, is committed to the well-being of patients.” “V *eut apprendre approche professionnelle, a à cœur le bien-être de ses patients.”*	1	0	0
“Great clerk orientation and supervision.” *“A très bien orienté et supervisé un externe.”*	0	1	0
“Takes initiatives in managing the patient” *“Prends l’initiative de la prise en charge”*	0	1	0
**Scholar role**			
“Emergency decisional algorithms are not always mastered (e.g., child with a fever), which places [the resident’s] knowledge at a below average level.” *Original French: “Algorithmes décisionnels en lien avec l’urgence ne sont pas toujours acquis (fièvre chez l’enfant...) ce qui le positionne en-dessous de la moyenne au niveau des connaissances.”*	1	1	1

Note. Original French version is in italic. R: Reliable; S: Specific; PI: Presence of pathways for improvement. 0 = absence of criterion; 1 = presence of criterion.

### Reliability

Interrater reliability was relatively solid regarding overall feedback quality in both the pre- (2013-2015) and the post-implementation (2016-2018) cohorts with intraclass correlations of .81 (p = .00) and .83 (p = .00), respectively. It was also very good for separate criteria with .73/.74 for Relevant (pre/post); .77/.79 for Specific; and .90/.92 for Pathways for Improvement. Internal consistency was also tested to determine whether the three feedback quality criteria are homogeneous and whether merging them into a single FQS score would be reliable. Internal consistency (Cronbach’s alpha) among the three quality scores (Reliability, Specificity and Pathways for Improvement) is.41 for the 400 reports; .36 for pre-implementation reports; and .46 for the post-implementation cohort, while the expectation is an alpha exceeding .70 ( Nunnally & Bernstein, 1994). Those low coefficients suggest that the three quality scores should be interpreted separately instead of with an aggregate FQS in the current study. In addition, PI is correlated to R with r = .18 (p = .01), and correlated to S with r = .30 (p = .00), while R is correlated to S with r = .41 (p = .00; see Table 4 in the section “correlations with other variables”).

### Cohort comparison

**Figure 1.**
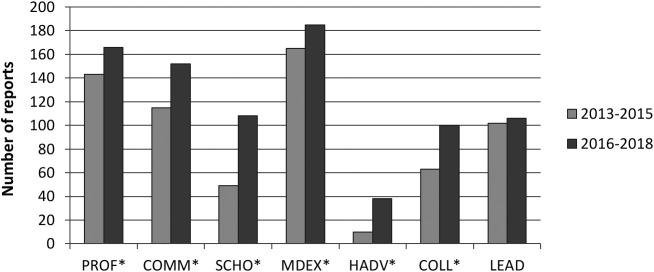
Number of reports containing comments about each CanMEDS role.

Note. *: p < .05; PROF = Professional; COMM = Communicator; SCHO = Scholar; MDEX = Medical expert; HADV = Health advocate; COLL = Collaborator; LEAD = Leader. Professionalism: χ ^2^(1) = 7.53, *p* = .01; Communicator χ ^2^(1) = 15.42, *p* = .00; Scholar χ ^2^(1) = 36.50, *p* = .00; Medical expert χ ^2^(1) = 9.14, *p* = .00; Health advocate χ ^2^(1) = 18.56, *p* = .00; Collaborator χ ^2^(1) = 14.18, *p* = .00; Leader χ ^2^(1) = 0.16, *p* = .69.

On average, pre-implementation report comments were provided 61.1% of the time (concerning five possible comment zones) and 58.6% of the time for post-implementation reports (concerning eight possible comment zones). However, this difference is not significant (t [393.47] = .95, p = .34). When comparing in terms of role coverage, the number of reports having covered a given role increased for all roles except Leader (see Figure 1).

Score comparison for ANOVA (see Table 2) revealed no difference whatsoever in feedback quality between cohorts. However, the difference between cohorts for relevance is marginally significant ( *F* = 3.52, *p* = 0.06). Feedback quality scores per criterion are illustrated on an aggregate basis, per rotation level, and per CanMEDS role in Figure 2. When compared to the CanMEDS role, ANOVA revealed that relevance diminished for Scholar in the post-implementation cohort ( *F* = 4.63, *p* = 0.03; η ^2^ = .03), as well as for Specificity for Scholar ( *F* = 13.63, *p* = 0.00; η ^2^ = .08); Health Advocate ( *F* = 4.48, *p* = 0.04; η ^2^ = .09); Collaborator ( *F* = 17.11, *p* = 0.00; η ^2^ = .10); and Leader ( *F* = 5.96, *p* = 0.02; η ^2^ = .03). According to Cohen’s rule of thumb (1988), eta-squared effect sizes of .02 are small and of .13 are medium. The other pre-post comparisons presented in Figure 2 are not statistically significant.

**Table 2.** Mean quality scores per cohort, cohorts differences in ANOVA, and CanMEDS coverage reports

	Cohort samples		Difference testing		
	2013-2015 (n=200)	2016-2018 (n = 200)	F	p	n2
**Mean Feedback Quality Score (SD)**					
**All (R1 & R2)**	65,1% (0.25)	67.6% (0.24)	0.99	.32	n.s.
**Relevance**	83.9% (0.25)	88.0% (0.18)	3.52	.06	n.s.
**Specificity**	69.2% (0.33)	69.0% (0.28)	0.01	.94	n.s.
**Pathways for improvement**	42.0% (0.99)	45.5% (0.50)	0.99	.32	n.s.
**R1**	65.1% (0.24)	70.4% (0.25)	1.98	.16	n.s.
**Relevance**	81.4% (0.27)	87.2% (0.20)	2.63	.11	n.s.
**Specificity**	67.4% (0.32)	66.6% (0.31)	0.07	.79	n.s.
**Pathways for improvement**	46.0% (0.50)	57.6% (0.50)	2.43	.12	n.s.
**R2**	65.1% (0.25)	65.1% (0.23)	0.00	.99	n.s.
**Relevance**	86.3% (0.23)	89.1% (0.16)	1.00	.32	n.s.
**Specificity**	71.1% (0.34)	71.8% (0.25)	0.03	.85	n.s.
**Pathways for improvement**	42.0% (0.49)	34.0% (0.48)	0.34	.56	n.s.
**Valence**	88.0% (0.22)	85.4% (0.26)	1.20	.27	n.s.
**R1**	87.0% (0.22)	78.6% (0.30)	4.67	.03	.15
**R2**	89.0% (0.21)	92.1% (0.18)	1.22	.27	n.s.

CanMEDS commentary	Number of reports with commentary	
Leader	143	166
Collaborator	115	152
Health Advocate	49	108
Medical expert	165	185
Scholar	10	38
Communicator	63	100
Professional	102	106

Note. Statistical difference is tested through ANOVA. p = probability (significance is noted with values <.05). When significant, η2 is provided, indicating effect size, which is interpreted as following: ~ .01: small effect; ~.09: medium effect; ~0.25: large effect.

**Figure 2a.**
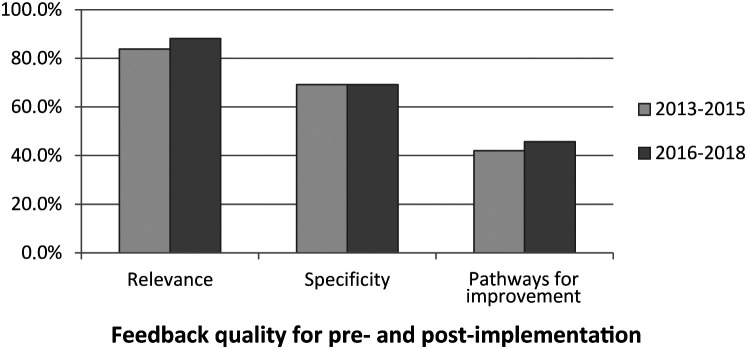
Feedback quality for pre (2013-2015) and post (2016-2018) CAT implementation

**Figure 2b.**
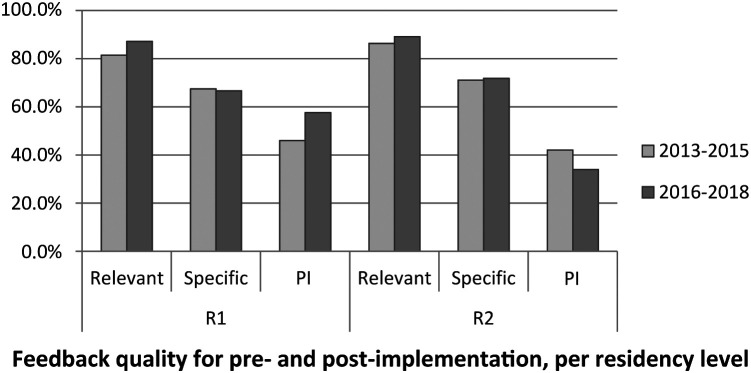
Feedback quality for pre (2013-2015) and post (2016-2018) CAT implementation, per residency level

Note. PI = Pathways for improvement.

**Figure 2c.**
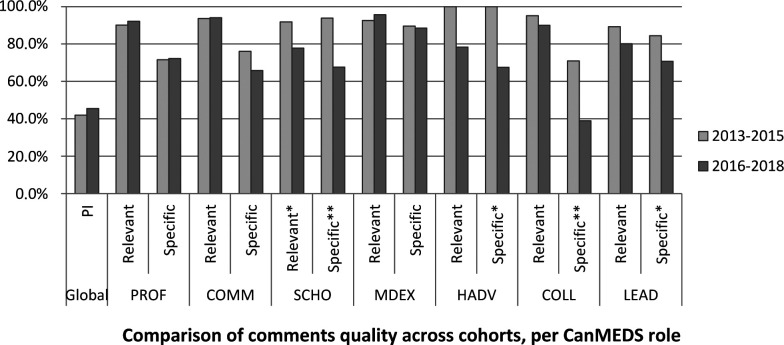
Comparison of comments quality across cohorts, per CanMEDs role

Note. *: p < .05; PROF = Professional; COMM = Communicator; SCHO = Scholar; MDEX = Medical expert; HADV = Health advocate; COLL = Collaborator; LEAD = Leader; PI = Pathways for improvement.

### Convergence with other variables

Correlations between total scores are presented in Table 3. Valence is only correlated with PI in the 2013-2015 cohort, and it is correlated with both PI and S but not with R in the 2016-2018 cohort. As for performance, the CAS is only correlated with PI.

**Table 3.** Correlations between feedback quality scores, feedback valence, and the competency acquisition score

	R	S	PI	Valence	% commented	PSC	CAS
**All cohorts**							
** Relevance**	-	.39 **	.04	-.04	-.06	-.10 *	
**Specificity**		-	.27 **	-.18 **	.05	-.17 **	
**PI**			-	-.59 **	.21 **	-.26 **	
**Valence**				-	-.07	.38 **	
**% commented**					-	-.01	
**PSC**						-	
**Pre-implementation**							
** Relevance**	-	.39 **	0.06	.04	-.07	-.12	
**Specificity**		-	.24 **	-.14	.08	-.16 *	
**PI**			-	-.63 **	.30 **	-.31 **	
**Valence**				-	-.21 **	.41 **	
**% commented**					-	.12	
**PSC**						-	
**Post-implementation**
** Relevance**	-	.41 **	.16	-.13	-.04	-.23 **	-.02
**Specificity**		-	.30 **	-.23 **	.01	-.23 **	-.07
**PI**			-	-.55 **	.10	-.33 **	-.43 **
**Valence**				-	.07	.54 **	.52 **
**% commented**					-	-.18*	-.02
** PSC**						-	.38 **

Note. R: Relevance, S: Specificity, PI: Presence of pathways for improvement, PSC: percentage of success in competencies, CAS: Competency acquisition score.

* p < .05

** p < .01

### Feedback quality and progress difficulties

Even though inferential statistics could not be computed between student groups, Table 4 shows mean quality scores for pre-CAT post-CAT implementation. There seems to be a tendency in both cohorts indicating that comments are more specific when students experience difficulties, and even more when there are progress concerns without failure. Before the CAT, the valence seemed to be drastically different if students experienced difficulties, a result that might be less radical with the CAT.

**Table 4.** Mean quality scores (in percentage) and standard deviation per cohort and residents’ difficulty level

Cohort		Residents’ performance		
Dependant variable		Expected	Progress concerns	Failure or in difficulty
**2013-2015**	**N =**	**185**	**7**	**8**
Relevance		83.67 (26.04)	92.43 (13.46)	81.38 (15.76)
Specificity		68.16 (34.21)	82.00 (13.60)	83.38 (19.38)
Pathways for improvement		37.30 (48.49)	1.00 (0.00)	1.00 (0.00)
Valence		92.22 (14.84)	39.00 (22.52)	34.38 (28.03)
PSC		61.01 (8.38)	48.00 (2.00)	43.50 (5.95)
**2016-2018**	**N =**	**159**	**16**	**25**
Relevance		86.57 (19.18)	96.25 (10.88)	92.04 (12.93)
Specificity		64.76 (29.45)	90.88 (11.47)	81.80 (18.41)
Pathways for improvement		44.94 (49.90)	50.00 (51.64)	48.00 (50.99)
Valence		95.34 (13.00)	53.50 (32.92)	42.88 (21.96)
PSC		69.14 (4.45)	63.44 (1.97)	56.80 (5.84)

Note. Progress concerns: students experiencing isolated competency achievement delays; Failure or in difficulty: based on the capstone.

### Rotation comparison

**Figure 3.**
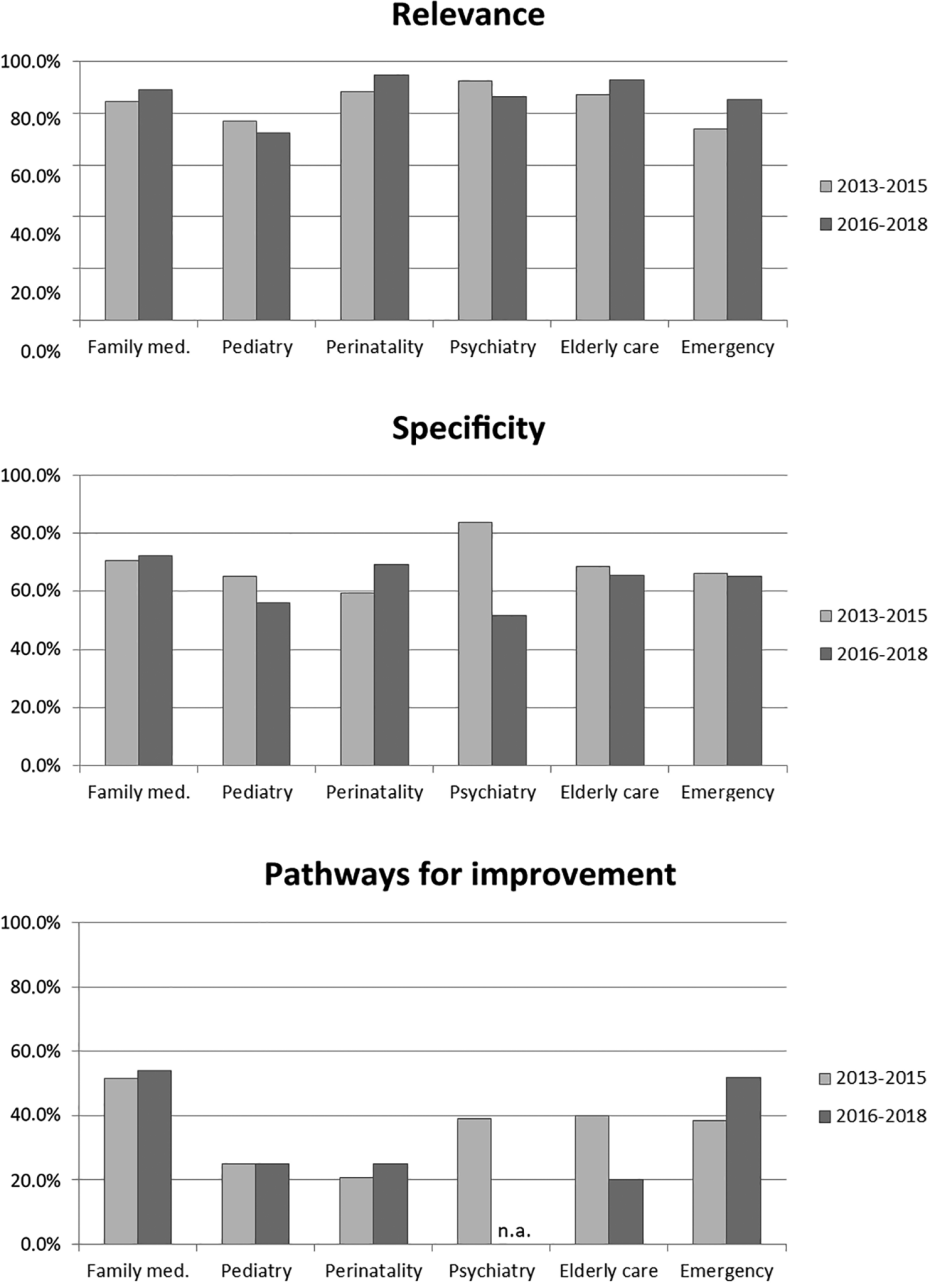
Mean feedback quality scores per program, for each cohort.

Rotation comparison concerning the three quality criteria is illustrated in Figure 3. In the ANOVA for the 2013-2015 cohort, there were no statistical differences in R, S, nor PI scores (Welsh’s F _R_ = 1.91, p = .11; F _S_ = 1.22, p = .30; Welsh’s F _PI_ = 2.38, p = .05), though the PI difference is marginally significant. Post-hoc contrasts highlight a large difference between family medicine and perinatality concerning PI (p = .04; Cohen’s d = .67). In the 2016-2018 cohort, the PI scores actually vary across programs (Welsh’s F _R_ = 1.72, p = .16; Welsh’s F _S_ = 1.06, p = .40; Welsh’s F _PI_ [excluding psychiatry, being without variance] = 3.16, p = .03). This time, psychiatry (where the scores for all eight reports were zero) differed significantly from family medicine rotations (p = .00; Cohen’s d = 1.53) and emergency (p = .00; Cohen’s d = 1.44).

## Discussion

The goal of the present study was to further validate the new CAT by verifying whether changes in the form and content of reports were associated with changes in feedback quality concerning the written comments accompanying the reports. We established three criteria to determine written feedback quality in the context of CAT administration: relevance, specificity, and the presence of pathways for improvement. Low homogeneity in scores among the criteria suggests that they should be interpreted separately instead of as an aggregate score. As for our analysis frame, agreement between the raters is good, while it is excellent for the category Pathways for Improvement, suggesting that the criteria were clearly and unanimously understood by the raters. The higher agreement for PI might be because the definition was simpler and required less interpretation for the coders.

Results indicate that the CAT does not appear to be associated with an increase in feedback quality. Most comments were rated as relevant in both cohorts: in 83.9% of the 2013-2015 reports and in 88.0% of the 2016-2018 reports (though relevance is marginally higher in the 2016-2018 cohort), a finding which is encouraging. Specificity was a little less common, emerging respectively in 69.2% and 69.0% of the reports. The addition of more details and examples in the new report may have taken the words out of their mouths, as it were, leaving the clinical teachers with not much to add. Furthermore, the specificity level differences are comparable across cohorts, suggesting that implementation of the new CAT might not have impacted specificity. In general, pathways for improvement are suggested in only 42.0% (2013-2015) and 45.7% (2016-2018) of reports, which also do not differ across cohorts. While the latter percentages may appear low, the residents are performing at a very high level: an “expected” level of performance corresponds to a PSC (percentage of success for the competencies) of 50% in 2013-2015 and of 66.67% in 2016-2018 and the means are 59.86% and 67.14%, respectively. In this context, it may be difficult for clinical teachers to find any improvement to suggest to such outstanding residents.

Clinical teachers completed the comments section with a similar amount of feedback before and after implementation of the CAT. As Lefroy *et al*. (2015) states, grades should not be provided without commentary; hence, we should expect to obtain comments covering the seven CanMEDS roles. In this matter, the new report provides better overall coverage of the various CanMEDS roles (excluding Leader), but Health Advocate is still the least covered role in both cohorts. The fact that CanMEDS are better covered with the CAT might be attributable to the explicit effort that was made during its development to clarify each role in detail with concrete rubrics. This might have facilitated the feedback provider’s role by reminding each provider of the aspects under assessment. Feedback quality is similar among cohorts, but when roles are taken separately, Scholar comments are less relevant and specific (small and medium-large effect, respectively); while specificity is reduced for Health Advocate (medium-large effect); Collaborator (medium-large effect); and Leader (small effect). When comparing the different rotations, clinical teachers in perinatality appeared to propose fewer pathways for improvement than in family medicine as regards the 2013-2015 cohort. Differences in PI scores also vary in general for the 2016-2018 cohort, with the eight reports in psychiatry not proposing any pathway for improvement whatsoever. While observation of the figures provided may suggest more differences, some samples are very small, reducing statistical power, thus suggesting that more investigation would be necessary before drawing further conclusions from these differences. Correlations suggest that lower performance is associated with more relevant and more specific comments (only with the PSC score), as well as more suggestions for improvement (and vice versa). Good performance is associated with positive comments, and poorer performance with negative comments. However, performance is not associated with the proportion of comments (i.e. the CanMEDS roles coverage).

Descriptive statistics contrasting groups based on their difficulties (in Table 4) reveal that comments are more specific when residents actually experience difficulties, and they are more relevant if the residents experience only some isolated difficulties (the “progress concern” group). Even if the sample size is too small to produce inferential statistics to compare cohorts, these preliminary results remain encouraging. Relevance seems similar among cohorts, while the increase of specificity for residents with some progress concern is even more distinguishable. Also, pathways for improvement appear to be proposed more often in the 2016-2018 cohort. Such an observation could be congruent with the new report’s aim, i.e. to support teachers’ feedback by providing explicit expectations and observable behaviours. This could help them target precise strengths and difficulties in their student’s performance. The phenomenon should definitely be further investigated in future research with larger samples of students with and without difficulties.

Some elements might have constituted limitations in the current study. Firstly, as the three criteria appear to be distinct from one another, it might be advisable to separate them into a few indicators, as they are constructs, not variables or objective realities ( Nunnally and Bernstein, 1994). This would improve reliability by broadening the raters’ understanding of those criteria, hence boosting inter-rater reliability, and also adding to the internal consistency of the scale. This nuance might seem technical but it is very important for an apposite interpretation of the R, S, and PI scores. For example, “relevance” is a rather interpretable term, even with the definition we provided. On the one hand, some indicators could be added, such as “relates explicitly to aspects of the CanMEDS definition;” “is based on facts and avoids generalities;” or “is based on remediable behaviors.” On the other hand, inter-rater reliability is very strong, suggesting that the criteria were understood across the board.

A second limitation is that the independence of observations could not be respected with our data, as clinical teachers and residents may be involved in one report or more. This can have the effect of artificially inflating the correlations or other analyses based on covariance. The third limitation relates to the feedback quality raters. Both cohort feedback quality criteria were coded by CS and LDB, but LC only coded the 2016-2018 reports. This resulted in a slight divergence in final score attribution and some limitations in the 2013-2015 triangulation of data. Fourth, the descriptive statistics about students in difficulty might suggest promising hypotheses, but the samples are too small to yield clear results or conclusions on the subject. In light of these limitations, the study could be improved in any replication, but none of the limitations constitutes a major obstacle to reaching the study objectives.

To the best of our knowledge, there was no framework adapted to written feedback accompanying periodical summative reports which could be applicable to our study. Therefore, we hope to have provided a basic explanation and breakdown of indicators to facilitate future feedback quality assessment and research on the subject. In addition to more specific indicators for each of the three criteria, this framework could also benefit from a comparison of the scores with the recipients’ point of view about the constructiveness of the feedback they received as well as whether or not they perceive it to be actually constructive and beneficial for learning. With a larger sample, norms could also be established in order to convert the scores into interpretable qualifiers and an expectation cut-off. While validity based on consequences of testing is supported for the new version of the CAT, further investigation is needed to develop a complete framework for assessing written feedback in the context of summative reports, which could help clinical teachers improve the quality of their own feedback. Indeed, the changes to the assessment and its report were numerous: 1) the competencies being measured were changed; 2) the measurement is now criterion-based, compared to the previous normative measurement; 3) the tool is now computerized, while it was previously on paper; and 4) competency indicators with examples are now provided. Because all the changes occurred at the same time across the program, it is impossible to isolate the contribution of each of these to the results we observe. While some changes might have improved feedback quality, some might have hindered it, resulting in only small observed changes. This limitation is not unusual in educational feedback studies in medical education; feedback is often confused with other interventions, as different changes to practices are implemented simultaneously ( Veloski *et al*., 2006). Also, few studies involve randomized educational trials in order to rigorously estimate the different approaches to feedback ( Bing-You *et al*., 2017). In the end, while some differences were observed among cohorts, none of them suggest that changes to the assessment of family medicine residents had substantive consequences for feedback quality, which was expected at the very least. Ideally, we would have hoped that the new CAT improved constructiveness of written comments, but this does not appear to be the case. As a complement, it would be interesting to investigate residents’ points of view about the quality of the feedback they received. Tools have been developed to look into the question (e.g. Bing-You *et al*., 2018). Though feedback quality was not affected from our point of view, it might be more appreciated and perceived as helpful by the residents themselves.

## Conclusion

We believe it is essential to take into consideration feedback quality as a consequence of testing in medical education when validating the implementation of new testing methods. Nevertheless, even if clinical teachers know how to draft and provide constructive feedback, many factors can influence feedback quality and the recipient’s adherence to the procedures that the feedback may suggest. The challenge for clinical teachers relates to all the engagement required to nurture the underlying dynamic process of feedback quality and educational alliance. It is essential for feedback providers and receivers to engage in a long-term relationship, where mutual respect is established, along with trust in the other’s good intent. It opens the door to free expression, both for the teacher who should feel comfortable in pointing out the learner’s difficulties, as well as for the learner in asking questions, expressing uncertainties, and communicating learning needs. In an era of computerization, there is a risk that such complex interaction might be less naturally established. In this context, continuing efforts must be made to support quality written and computerized feedback practices.

## Take Home Messages

• The literature suggests that when feedback is delivered with periodic written reports, feedback quality is determined by relevance, specificity, and the presence of pathways for improvement.
• Feedback quality did not change with the implementation of a criterion-based, computerized, and summative assessment report.
• Computerized feedback aimed at supporting clinical teachers’ decisions with examples appears to contribute to better coverage of the various CanMEDS roles.
• Comments appear to be more specific for learners in difficulty. When learners only experience some difficulties without being at risk of failure, feedback appears to be even more specific.

## Notes On Contributors

**Caroline Simard**, PhD, was at the time of the study a research professional in medical education at the Department of Family Medicine and Emergency Medicine, Université Laval, Quebec City, Canada. ORCID ID: https://orcid.org/0000-0002-3545-8041

**Luc Côté**, MSW, PhD (ed.) is professor at the Department of Family Medicine and Emergency Medicine, Université Laval, Quebec City, Canada.

**Laurie de Bruyn,** was at the time of the study a medical student, Université Laval, Quebec City, Canada.

**Miriam Lacasse**, MD MSc CCFP, is a family physician and associate professor at the Department of Family Medicine and Emergency Medicine, Université Laval, Quebec City, Canada. She co-chairs the Educational Leadership Chair in Health Professions Education and is evaluation director for the family medicine residency program. ORCID ID: https://orcid.org/0000-0002-2981-0942
