# Supplementary material for: CanMEDS Competencies in Family Medicine Residents: Can Criterion-Based Assessment Improve the Quality of Teacher Feedback?
Source: MedEdPublish (2016). 2021 Jan 19;10:16. [Version 1] doi: 10.15694/mep.2021.000016.1 (PMC10939571; doi:10.15694/mep.2021.000016.1)
Supplement: Peer review history

# Reviewer report: Reviewer response for version 1

**DOI:** 10.21956/mep.18868.r26916

**Published:** 2021-04-16

**Authors:** Ken Masters

**Affiliations:** 1 Sultan Qaboos University

This review has been migrated. The reviewer awarded 4 stars out of 5 A really interesting and solidly-written paper on criterion-based assessment and the quality of medical teacher feedback.The authors have given a useful and detailed background on the issue of feedback. I really do like the feedback summary given in the paper’s Introduction. That could probably serve as useful stimulus material for a staff development workshop. The results are displayed in great detail.Some issues that need to be addressed:• The Abstract’s background needs to explain a little about what the tool is designed to do. The Abstract’s introduction is extremely short, and we learn only that it is a “criterion-based Competency Assessment Tool”. Because this is so broad, and could apply to almost anything, the rest of the Abstract is a fog. This, combined with other non-specific deals (e.g. “the cohorts”) means that most readers will finish the abstract without actually knowing much about the work. Please revise the Abstract (It might be a good idea to have the Abstract reviewed by someone not familiar with the work or context.)• Similarly, the aim of the research should be given in the Abstract.• A link should be provided to the online tool. (If it is not available for public use, then this should be mentioned).• “The consensus is well established in medical education concerning what constitutes effective feedback” Mmm. I’m not so sure about that. I’m not going to push this point, but the authors really might consider re-assessing their confidence in that statement.• I’m disappointed that only one “Scholar role” example was given, and would like to see more. This would allow the reader to have a greater sense of the scope of the responses. Smaller issues:• The paragraph under “Quantitative data analysis” is a massive wall of text, and could benefit from being broken into small paragraphs. Some other large paragraphs could similarly be broken up a little.• In Table 1, in the last comment, the “Original French:” title could be removed, as the explanation of the italics is given in the key.• Table 4, last two rows: The alignment needs adjusting.So, a really strong paper, but I would like to see Version 2 addressing these points.

Reviewer Expertise:

NA

No decision status is available

# Reviewer report: Reviewer response for version 1

**DOI:** 10.21956/mep.18868.r26915

**Published:** 2021-01-26

**Authors:** David Bruce

**Affiliations:** 1 None

This review has been migrated. The reviewer awarded 4 stars out of 5 I think the authors are to be congratulated for this thorough and detailed paper on how the introduction their new assessment tool affects the quality of feedback that the residents on their family medicine programme receive. The Competency Assessment Tool (CAT) was developed from the CanMEDS framework and was an on-line document that guided the teachers to provide feedback for each of the CanMEDS competencies. While the quality of feedback was not improved with the introduction of this tool, feedback comments covered a wider range of competencies. The authors set out their ideas very clearly in the introduction stating the need to consider the consequential validity of an assessment tool. This is one part of the utility of an assessment tool and it’s good to see it being considered in this detail. I thought the review of feedback provided was excellent. As a minor point I wondered if the sentence - learner's perception of his or her trainee's authenticity – should read the teachers perception? The methodology for scoring the feedback quality was outlined and again demonstrated the thoroughness of the authors approach. Some more detail about how feedback was provided in the pre-CAT group would have been helpful – I was not clear if this was also a structured approach or just free text comments. The context of the feedback quality which correlated the three feedback criteria with the assessment of the valence of the comments and the performance score was difficult to follow as a non-expert reader. The results are clearly set out in full detail with good use of graphs and tables. Finally, the authors consider the results and (in my view anyway) perhaps understate the importance of their findings. The new tool facilitates comments in each of the CanMEDS competencies while not decreasing any of the feedback quality the residents receive. I did wonder if providing an on-line tool that guided the teachers into making comments that cover the all the CanMEDS competencies might have resulted in less specific, relevant comments and less suggestions for improvement – so I would see the results in a more positive light. Also, of note is the finding that feedback quality remains high in the key groups of residents who are struggling and not progressing as their teachers would like. An excellent paper. I wondered if a summary version for non-expert readers would be helpful to increase the impact of the authors message.

Reviewer Expertise:

NA

No decision status is available

# Reviewer report: Reviewer response for version 1

**DOI:** 10.21956/mep.18868.r26914

**Published:** 2021-01-21

**Authors:** Alice Fornari

**Affiliations:** 1 Donald and Barbara Zucker School of Medicine at Hofstra/Northwell

This review has been migrated. The reviewer awarded 4 stars out of 5 Thank you for addressing in your research an ongoing struggle in medical education, quality feedback. connection to a conceptual framework like CanMeds is valuable.

Reviewer Expertise:

NA

No decision status is available
